# Maternal age and educational level modify the association between chronic hepatitis B infection and preterm labor

**DOI:** 10.1186/s12884-020-2729-1

**Published:** 2020-01-14

**Authors:** Songxu Peng, Hongyan Chen, Xiu Li, Yukai Du, Yong Gan

**Affiliations:** 1 Department of Maternal and Child Health, Xiangya School of Public Health, Central South University, Changsha, China; 2 Department of Maternal and Child Health, School of Public Health, Tongji Medical College, Huazhong University of Science and Technology, 13th Hangkong Road, Wuhan, Hubei China; 3 Department of Social Medicine and Health Management, School of Public Health, Tongji Medical College, Huazhong University of Science and Technology, No. 13 Hangkong Road, Wuhan, 430030 China

**Keywords:** Hepatitis B virus infection, Preterm labor, Maternal age, Educational level

## Abstract

**Background:**

Few studies have investigated whether maternal age and education level modify the association of chronic hepatitis B virus (HBV) infection with preterm labor. We hypothesized that the association of HBV infection with preterm labor is modified by maternal age and education level.

**Methods:**

A retrospective cohort study was conducted on the HBsAg-positive and HBsAg-negative pregnant women delivered from June 2012 to August 2017 at Wuhan Medical Care Center for Women and Children, Wuhan, China. A multivariate regression model was used in this study.

**Results:**

This study included 2050 HBsAg-positive pregnant women and 2050 HBsAg negative women. In the stratified analyses, positive HBsAg status was associated with the increased risk of preterm labor in women aged < 30 years, having low educational level, with an odds ratio of 1.65(95% CI 1.07–2.54) and 2.59(95% CI 1.41–4.76), respectively. Breslow-Day test showed that there existed significant differences in the ORs for HBsAg carriage across each stratum of maternal age (*p* = 0.023), educational level (*p* = 0.002). After adjusting other co-variables, we observed maternal HBV infection (OR 1.60, 95% CI 1.03–2.49) was still associated with risk of preterm labor in pregnancy women with age < 30. Similarly, the significant association of HBV infection (OR 2.49, 95% CI 1.34–4.63) with preterm labor remained in low educated women.

**Conclusions:**

Our results indicated that HBV infection was associated with high risk of preterm labor, but maternal age and educational level could modify the association between HBV infection and preterm labor.

## Background

Hepatitis B virus (HBV) infection is one of the most common health problems, causing high mortality and heavy economic burden worldwide [1–3]. With approximately 350 million chronic HBV patients around the world, almost one third of chronic HBV carriers live in China [4]. Therefore, China has the world’s largest burden of hepatitis B virus infection [5]. The prevalence of HBV infection in the general population at different ages varies widely in China [5, 6]. The women of childbearing age have been estimated at 5.2–6.7% [7, 8], which are the main source of hepatitis B transmission. However, most pregnant women with HBV infection are chronic carriers [9], indicated by positive serum hepatitis B surface antigen (HBsAg) status. Therefore, whether HBV carriers could negatively influence pregnancy outcomes becomes a critical issue.

Preterm labor (PTB, delivery prior to 37 weeks’ gestation) is the leading cause of neonatal morbidity and mortality in high-resource countries [10]. Its complications are estimated to account for approximately 35% of the neonatal deaths annually [11], and surviving preterm babies have an increased risk of neurodevelopmental impairments, respiratory, and gastrointestinal complications [12, 13]. Findings from previous studies show that preterm labor is associated with several maternal risk factors [14], pregnancy history and characteristics, as well as many genetic, environmental, and societal factors [15, 16]. Among these factors, maternal viral infection is an important risk factor for preterm labor, mainly due to the activation of inflammatory pathways by viral factors [17, 18]. A large number of studies have explored the impact of maternal chronic HBV infection on preterm labor, but the research results are inconsistent. Some studies suggest maternal HBV infection is associated with an increased risk of preterm labor [19–21]. However, other studies find no such association [22, 23].

Maternal age and education level are general demographic characteristics, most frequently studied as factors influencing health. Maternal age and education level are common factors that have been related both to HBV infection and preterm labor [24–26]. The accumulated evidence indicated that the population with different ages had various degrees of infection risk after exposure to hepatitis B virus [27, 28]. Even in women with HBV infection, age is associated with HBV DNA level and hepatitis B e antigen (HBeAg) status. Young women are more likely to have a high HBV viral load and HBeAg positivity than older women [29]. Additionally, the effects of maternal age on preterm labor have long been reported. Advanced maternal age significantly increased the risk of preterm labor [30]. Similarly, maternal education level may influence HBV infection status and preterm labor risk. Low schooling level is significantly associated with HBV infection and preterm labor [25, 26]. Whether maternal age and educational level could modify the relationship between HBV infection and preterm labor is unclear.

In this hospital-based retrospective cohort study, we investigated whether maternal age and education level could modify the association of chronic HBV infection with preterm labor.

## Methods

### Study population

A hospital-based retrospective cohort study was performed on singleton pregnancies delivered from June 2012 to August 2017 at Wuhan Medical Care Center for Women and Children, Wuhan, China. The annual delivery rate of this hospital was around 5000 live births. The majority of the parturients were local residents, > 98% of whom are ethnic Han. All pregnant women received a routine screening for HBsAg and the antibodies to HCV, HIV and TP by enzyme-linked immunosorbent assay (ELISA) at the first antenatal visit. All HBsAg-positive women aged 18 or older with singleton pregnancy, who had no current or previous medical complications (including diabetes mellitus, hypertension, psychiatric illness, HCV, HIV or TP infection), were assigned to HBsAg-positive group. A total of 2050 HBsAg-positive women were eligible for the study. For each case, a control without HBsAg, matched for age and parity, was identified and randomly chosen from electronic databases using the same criteria mentioned.

The present study was approved by the Institutional Review Board of Tongji Medical College, Huazhong University of Science and Technology. The written informed consent of all subjects was obtained before participation in this study.

### Data collection and samples collection

The clinical records of participants in the two groups were retrieved for data extraction. The maternal demographic and clinical information, including age, ethnicity, educational level, height, pre-pregnancy weight, gestational weight gain (GWG), history of pregnancy (including gravidity, parity, miscarriage, induced abortion and stillbirth), and gestational age were obtained from the obstetric records. Pre-pregnancy body mass index (BMI) was calculated as the ratio of pre-pregnancy weight (kg) divided by height (m^2^). Women with years of education ≤9 were considered having low educational level. Women with > 9 were classified as having high educational level. Gestational age was based on the interval between the date of last menstrual period and the date of delivery. Preterm labor was defined as delivery with gestational age of less than 37.

### Statistical analysis

Continuous variables were expressed as the mean ± standard deviation (SD) and analyzed by Student’s t-tests. Categorical data were expressed as percentages and compared by chi-square tests. Stratified analyses were used to identify effects of maternal age and educational level on the association between HBV infection and preterm labor. The homogeneity of the odds ratios for HBV infection across each stratum of age and educational level was assessed by Breslow-Day test. Using a likelihood ratio test in the logistic regression model, multiplicative interaction was tested to evaluate interactions between maternal age, educational level, and HBV infection. Multivariable logistical regression was used to measure the independent association between HBV infection and preterm labor stratified by maternal age and education level. Statistical significance was assessed at *p* < 0.05 (two-tail test). All analyses were performed using SPSS software version 18.0 (SPSS, Chicago, IL, USA).

## Results

In total 4100 women were included in the analysis with 2050 HBsAg-positive women and 2050 HBsAg-negative women identified during June 2012 to August 2017. The characteristics of two groups are showed in Table 1. The distribution of maternal education level was significantly different between the two groups. HBsAg-positive women were more likely to have a high educational level, compared with HBsAg-negative mothers. But there were no significant differences in maternal age, ethnicity, pre-pregnancy BMI, gestational weight gain (GWG), gravidity, parity, history of miscarriage, history of induced abortion, or history of stillbirth between HBsAg-positive group and HBsAg-negative group.

**Table 1:** Maternal characteristics and clinical features by HBsAg status among the participants

Characteristics	HBsAg + (*n* = 2050)	HBsAg - (*n* = 2050)	*P*-value
Age (years, mean ± SD)	29.0 ± 4.1	29.0 ± 4.1	1.000
Age < 30	1271(62.0)	1271(62.0)	1.000
Han nationality	2040(99.5)	2038(99.4)	0.669
Education			**< 0.001**
Low **e**ducational level	344(16.8)	461(22.5)	
High educational level	1706(83.2)	1589(77.5)	
Pre-pregnancy BMI	20.7 ± 2.6	20.9 ± 2.7	0.311
GWG	16.6 ± 6.2	16.8 ± 7.6	0.363
History of pregnancy			
History of stillbirth	6 (0.3)	5 (0.2)	0.763
History of miscarriage	68(3.3)	86(4.2)	0.139
History of induced abortion	324(15.8)	357(17.4)	0.166
First gestation	1010 (49.3)	1046(51.0)	0.261
Nulliparous	1616(78.8)	1616(78.8)	1.000
Preterm labor	108(5.3)	96(4.7)	0.389

*Abbreviation*: *SD* Standard deviation, *GWG* Gestational weight gain

To determine the interaction between maternal age, education level and HBsAg status, stratified analyses by HBsAg status were performed according to maternal age, educational level, i.e. age ≥ or < 30 years, educational level classified as low educational level, or high educational level. As shown in Table 2, positive HBsAg status was associated with the increased risk of preterm labor in women aged < 30 years, with an odds ratio (OR) of 1.65(95% CI 1.07–2.54). Similarly, an increased risk of preterm labor was observed among positive HBsAg women having low educational level (OR 2.59, 95% CI 1.41–4.76). However, this positive association vanished in women with age ≥ 30 or high educational level. Subsequently, the Breslow-Day test showed that there existed significant differences in the ORs for HBsAg carriage across each stratum of maternal age (*p* = 0.023), educational level (*p* = 0.002). In addition, multiplicative interactions between maternal age (*p* = 0.039), educational level (*p* = 0.003) and HBV infection was identified in the logistic regression model after adjusting for age, education level, pre-pregnancy BMI, GWG, HBsAg status.

**Table 2:** Incidence of preterm labor with respect to HBsAg status in pregnant women, stratified by maternal age, education level

Factors	PT (%)		OR(95% CI)	*P*-values^a^	*P*_mul_
	HBsAg +	HBsAg -			
Age					
< 30	4.3(55/1271)	2.7(34/1271)	1.65(1.07–2.54)	**0.023**	**0.039**
≥ 30	6.8(53/779)	8.0(62/779)	0.84(0.58–1.24)		
Education					
Low **e**ducational level	9.0(31/344)	3.7(17/461)	2.59(1.41–4.76)	**0.002**	**0.003**
High educational level	4.5(77/1706)	5.0(79/1589)	0.90(0.66–1.24)		

*P*_mul_ for multiplicative interaction between each risk factor and HBsAg status on PT, adjusting age, education level, pre-pregnancy BMI, GWG, HBsAg status

*Abbreviation*: *PT* Preterm labor, *OR* Odds ratio, *CI* Confidence interval

^a^*P*-values for interaction effect between each risk factor and HBsAg status on PT

Because the interactions between maternal age, educational level and HBsAg carrier were identified in stratified analyses. Multiple regressions between HBsAg status and preterm labor stratified by maternal age and education level were also performed. HBsAg and other risk factors (including maternal age and educational level as categorical variable, pre-pregnancy BMI and GWG as continuous variable) affecting the presence of preterm labor, were included into multivariable logistical regression analyses. Among the women with age < 30, HBsAg carriers (OR 1.61, 95% CI 1.04–2.51) were associated with the increased incidence of preterm labor, after adjustment for other associated covariates (Table 3). Women with high educational level (OR 0.60, 95% CI 0.37–0.97) had a decreased risk of preterm labor. In another subgroup with age ≥ 30, the association between HBV carrier and preterm labor did not reach significance.

**Table 3:** Multiple regressions between HBsAg status and preterm labor stratified by maternal age

Variable	Age < 30		Age ≥ 30	
	*OR (95% CI)*	*P*-value	*OR (95% CI)*	*P*-value
HBsAg	1.60 (1.03–2.49)	**0.036**	0.83 (0.57–1.22)	0.346
Education level	0.60 (0.37–0.97)	**0.037**	1.08 (0.67–1.76)	0.746
Pre-pregnancy BMI	1.06 (0.97–1.15)	0.200	1.02 (0.96–1.09)	0.486
GWG	0.97 (0.93–1.00)	0.058	0.99 (0.96–1.02)	0.332

*Abbreviation*: *OR* Odds ratio, *CI* Confidence interval, *BMI* Body mass index, *GWG* Gestational weight gain

Stratified by maternal education level, a significant association of maternal HBsAg carriage with the incidence of preterm labor was observed (OR 2.49, 95% CI 1.34–4.63) (Table 4) in the low educational level group. However, the association between HBV carriers and preterm labor vanished in the high educational level group. But in the high educational level group, the results of analyses showed that older pregnant women with age ≥ 30 (OR 2.53, 95% CI 1.81–3.53) had an increased risk of preterm labor compared with young women aged < 30. Simultaneously, significant association between GWG and decreased risk of preterm labor were detected in the high educational level group, with an OR value of 0.96 (95% CI 0.94–0.99).

**Table 4:** Multiple regressions between HBsAg status and preterm labor stratified by maternal educational level

Variable	Low educational level		High educational level	
	*OR (95% CI)*	*P*-value	*OR (95% CI)*	*P*-value
HBsAg	2.49 (1.34–4.63)	**0.004**	0.88 (0.63–1.21)	0.427
Age	1.46 (0.80–2.69)	0.222	2.53 (1.81–3.53)	**< 0.001**
Pre-pregnancy BMI	1.11 (0.99–1.25)	0.070	1.01 (0.96–1.07)	0.677
GWG	1.03 (0.98–1.07)	0.223	0.96 (0.94–0.99)	**0.004**

*Abbreviation*: *OR* Odds ratio, *CI* Confidence interval, *BMI* Body mass index, *GWG* Gestational weight gain

## Discussion

Our study found that there existed interactions between the maternal age, educational level and HBV infection and an association between HBV infection and preterm labor across different maternal age and education levels was observed. Maternal age and education levels may mediate the association between chronic hepatitis B infection and preterm labor.

In the present study, we found that maternal age, educational level, and GWG were associated with the presence of preterm labor in the partial subgroup analyses despite the fact that the associations did not always exist. We point that preterm labor is multifactorial, influenced by maternal characteristics and resulting in a differential risk of preterm labor in various subgroups.

The association between maternal HBV infection and preterm labor has been extensively studied over the past decades [19, 21–23], and modest positive associations have been reported in several large cohort studies [20]. Our study showed that HBV infection was associated with high risk of preterm labor in young or low educated women. Findings from previous studies have suggested that factors related to systemic inflammatory responses, such as liver injury (hepatitis, cirrhosis and hepatocellular carcinoma), that promote release of proinflammatory cytokines, have been considered as possible mechanisms for the observed association [31, 32]. Additionally, the long-term accumulation of HBV DNA in the placenta and trophoblast cells activated the placental inflammatory response and impaired trophoblasts and placental function [33, 34]. This could play a role in the link between HBV infection and preterm labor.

From the stratified analyses, our data suggest a modifying effect of maternal age and education level on the association between HBV infection and preterm labor in the present study. We found that among pregnant women with age < 30, HBV infection significantly increased the risk of preterm labor compared with the healthy group. Similar results could be seen in the low education level group. This positive association between HBV infection and preterm labor disappeared in the groups of older or highly educated pregnant women. The previous study demonstrated that advanced maternal ages were associated with hepatitis B virus load in pregnant women [30]. Elderly pregnant women tend to have low viral load [29], resulting in relatively mild inflammatory reaction. Additionally, hepatitis B virus DNA load was positively associated with HBV infection rates in the placental cell layers [35]. This explains to a certain extent why the association between HBV infection and preterm labor vanished in the older pregnancy women. A published study showed that educational differences of pregnant women may reflect differences in the way that women utilize health care systems [36]. Generally, the pregnant women with high educational level were positively associated with increased income, which is an important factor for accessibility of high-quality medical services. Moreover, educationally disadvantaged women could be more marginalised and vulnerable in societies compared with highly educated people. Consequently, pregnant women with low education could suffer from more stress in life and work [36]. There may be alternative explanations for a modifying effect of educational level on the associations between HBV infection and preterm labor.

This study has multiple strengths. First, the present study included a large number of subjects, which makes it possible to explore modification effects of external variables with confounding variables controlled in a prospective cohort study. Second, this study comprehensively explored the associations between HBV infection and preterm labor in different levels of maternal age and education, which provided more evidence for presentation of the targeted preventive strategies for pregnant women with different characteristics. Third, to our knowledge, this is the first study that has examined the potential modification effects of maternal variables on the association between HBV infection and preterm labor. However, the limitations of our study are unavoidable. The first and most obvious limitation is that our study is a retrospective study that proves a positive correlation between HBV infection and preterm labor. Thus, its capability of etiological inferences is limited. Therefore, a large-scale prospective study on this causal relationship is needed. Second, we did not collect the data of liver injury including hepatitis, cirrhosis and hepatocellular carcinoma in this study, which would have provided evidence for a positive association between HBV infection and preterm labor.

## Conclusion

HBV infection is associated with high risk of preterm labor. Advanced maternal age and high educational level could buffer the adverse association between maternal HBV infection and preterm labor. Unfortunately, the apparent moderating effect of maternal age and education level disappears at the young or low educational women. In clinical practice, according to risk stratification, targeted intervention should be taken in subgroup population, such as that HBV DNA level should be tested in pregnancy women with age < 30 or in low educated women.

## Data Availability

The datasets generated and/or analysed during the current study are not publicly available due to the research still being carried on but are available from the corresponding author on reasonable request.

## Abbreviations

BMI: Body mass index
CI: Confidence interval
ELISA: Enzyme-linked immunosorbent assay
GWG: Gestational weight gain
HBeAg: Hepatitis B e antigen
HBsAg: Hepatitis B surface antigen
HBV: Hepatitis B virus
HCV: Hepatitis C virus
HIV: Human immunodeficiency virus
OR: Odds ratio
PTB: Preterm labor
SD: Standard deviation
TP: *treponema pallidum*
